# A Forward Genetic Screen Reveals that Calcium-dependent Protein Kinase 3 Regulates Egress in *Toxoplasma*

**DOI:** 10.1371/journal.ppat.1003049

**Published:** 2012-11-29

**Authors:** Erin Garrison, Moritz Treeck, Emma Ehret, Heidi Butz, Tamila Garbuz, Benji P. Oswald, Matt Settles, John Boothroyd, Gustavo Arrizabalaga

**Affiliations:** 1 University of Idaho, Department of Biological Sciences, Moscow, Idaho, United States of America; 2 Stanford University School of Medicine, Department of Microbiology and Immunology, Stanford, California, United States of America; 3 Indiana University School of Medicine, Department of Pharmacology and Toxicology, Indianapolis, Indiana, United States of America; 4 University of Idaho, The Institute for Bioinformatics and Evolutionary Studies, Moscow, Idaho, United States of America; University of Geneva, Switzerland

## Abstract

Egress from the host cell is a crucial and highly regulated step in the biology of the obligate intracellular parasite, *Toxoplasma gondii*. Active egress depends on calcium fluxes and appears to be a crucial step in escaping the attack from the immune system and, potentially, in enabling the parasites to shuttle into appropriate cells for entry into the brain of the host. Previous genetic screens have yielded mutants defective in both ionophore-induced egress and ionophore-induced death. Using whole genome sequencing of one mutant and subsequent analysis of all mutants from these screens, we find that, remarkably, four independent mutants harbor a mis-sense mutation in the same gene, *TgCDPK3*, encoding a calcium-dependent protein kinase. All four mutations are predicted to alter key regions of TgCDPK3 and this is confirmed by biochemical studies of recombinant forms of each. By complementation we confirm a crucial role for TgCDPK3 in the rapid induction of parasite egress and we establish that TgCDPK3 is critical for formation of latent stages in the brains of mice. Genetic knockout of TgCDPK3 confirms a crucial role for this kinase in parasite egress and a non-essential role for it in the lytic cycle.

## Author Summary

*Toxoplasma gondii*, an important human pathogen, is an obligate intracellular parasite, thus getting in and out of cells is key for its survival. The process by which *Toxoplasma* exits cells, known as egress, is controlled by calcium fluxes and can be triggered by ionophores. *In vivo*, rapid egress from the host cell has been identified as a means to escape attack by the innate immune system. At the molecular level, calcium dependent events in this parasite are regulated in part by plant like calcium dependent kinases, which share no homology to human kinases and are thus ideal drug targets. In this study we revisited 4 mutant parasite lines that were independently selected for an inability to undergo egress in response to ionophores. In all four mutants we have identified the Calcium Dependent Kinase 3 as the gene responsible for the defects. We have shown that two of these mutants, which are in a genetic background that allows virulence studies, also have a strong phenotype *in vivo*. That is, the parasites fail to form latent stages in mice. This work provides important information that a single kinase is responsible for the formation of latent stages that are important for transmission of the parasite.

## Introduction

The obligate intracellular parasite *Toxoplasma gondii* chronically infects a third of the world's human population. While most infections are asymptomatic, in immunocompromised individuals such as those with AIDS, leukemia and lymphoma, new infections or rupture of pre-existing latent cysts can lead to toxoplasmic encephalitis [1]–[3]. Additionally, in congenital infections, the disease can lead to severe neurological problems or even death of the developing fetus [4].

Propagation within an infected host along with some of the dire consequences of an uncontrolled *T. gondii* infection are a direct result of its lytic cycle, which includes attachment to a host cell, invasion and egress [5]. The secretion of adhesins involved in attachment and the gliding motility of the parasite [6]–[8] and of perforins needed for egress [9], as well as the cytoskeletal rearrangements seen during *T. gondii* motility, invasion and egress [10] have been shown to be dependent on Ca^2+^ signaling [11]. The calcium critical for initiation of motility is released from intracellular compartments [12] and calcium regulated proteins such as calmodulin, centrins and calcium-dependent kinases (CDPKs) play important signaling roles [13]. In particular, TgCDPK1 has been shown to be upstream of a signaling pathway regulating microneme secretion during invasion and egress [14]. The relation between calcium fluxes and events involved in motility, invasion and egress is particularly evident in experiments with the calcium ionophore A23187. This ionophore induces intracellular parasites to become motile and exit their host cell in a process known as ionophore-induced egress (iiEgress) [15]. Similarly, when exposed to A23187, extracellular parasites activate the secretory and cytoskeletal events required for invasion [10], [16]. Prolonged ionophore exposure while extracellular causes *T. gondii* to irreversibly lose its ability to invade host cells, presumably due to the exhaustion of essential invasion factors [10]. This inhibition causes the parasite to die, and therefore is referred to as ionophore-induced death (iiDeath) [10]. Thus, both of these phenomena, iiEgress and iiDeath, can be used to dissect the processes that lead to the calcium-dependent initiation of motility, invasion and egress.

To study the signaling events involved in the parasite's response to calcium fluxes, we took a genetic approach by designing a screen for mutant parasites, generated with N-ethyl-N-nitrosourea (ENU), unable to exit the host cell after induction of egress with the ionophore A23187 [17]. Two independent mutant lines, MBE1.1 and MBE3.1, were established in this manner. While >95% of wild-type vacuoles are lysed after two minutes of exposure to A23187, less than 5% of mutant vacuoles were lysed at the same time point [17]. Importantly, unlike the egress phenotype in the TgCDPK1 knockdown strain, microneme secretion and gliding motility were not found to be impaired in these mutants when extracellular. Nonetheless, these mutants were shown to have a delay in parasite-dependent permeabilization of the host cell membrane [17] an event mediated by the micronemal perforin-like protein TgPLP1 [9]. A phenotypic difference between the MBE1.1 and the MBE3.1 strains was observed when testing for iiDeath: while MBE3.1 showed a normal iiDeath phenotype (i.e. parasites die after prolonged exposure to A23187), MBE1.1 parasites were significantly resistant [17]. To investigate the possibility that iiEgress and iiDeath are genetically related phenomena, a second selection was performed for parasites that survive extracellular A23187 treatment. Two mutant lines with iiDeath resistance were established in this manner, one of which also shows a severe delay in iiEgress (MBD1.1) and one that shows normal iiEgress (MBD2.1) (Table 1) [17]. More recently, we identified a mutant, 52F11, with both a delay in induced egress and extracellular resistance to the ionophore by screening through a signature-tagged library of mutants for those with an iiEgress phenotype [18]. Besides its ionophore-dependent phenotypes, 52F11 was less virulent than its parental strain in mice. Interestingly, a second independent clone from the same library, 91E4, originally selected for a reduction in virulence in mice, also exhibits a delay in iiEgress [18]. The fact that two independent strains show both a defect in iiEgress and *in vivo* cyst formation indicate that these two phenotypes are likely genotypically connected. Thus, in total we have a collection of six independent mutants that fall into three phenotypic categories: defective in both iiEgress and iiDeath (MBE1.1, MBD1.1 and 52F11, 91E4), defective in only iiEgress (MBE3.3) and defective in only iiDeath (MBD2.1) (Table 1).

**Table 1:** Mutants with defects in either iiEgress, iiDeath or both are listed along with a description of how they were selected or screened for and the mutation found in TgCDPK3.

Mutant	Parental strain	Method of isolation	Defect in:	Mutation in TgCDPK3
MBE1.1	RHΔ*hpt*	Selection for parasites defective in iiEgress	iiEgress and iiDeath	T239I
MBE3.3	RHΔ*hpt*	Selection for parasites defective in iiEgress	iiEgress	None
MBD1.1	RHΔ*hpt*	Selection for parasites defective in iiDeath	iiEgress and iiDeath	L184P
MBD2.1	RHΔ*hpt*	Selection for parasites defective in iiDeath	iiDeath	None
52F11	PruΔ*hpt*	Screen for parasites defective in iiEgress	iiEgress and iiDeath	G88D
91E4	PruΔ*hpt*	Screen for parasites with reduction in virulence	iiEgress and iiDeath	N204K

Defect in iiEgress is a delay in egress in response to calcium ionophore treatment and defect in iiDeath is a resistance to extracellular exposure to calcium ionophore.

The independent isolation of 4 mutants that have both iiEgress and iiDeath defects supports the idea that these two processes are genetically connected [19]. Whereas most data regarding ionophore-induced egress comes from studies performed in cell culture, there are strong indications that it might play an important role *in vivo*. *In vivo* and *ex vivo* it has been shown that *Toxoplasma* egress can be triggered during interaction with immune-effector cells in a perforin-, fas- and/or antigen-dependent manner [20], [21]. Intriguingly, rapid egress is often coupled with immediate invasion of the attacking immune cell. Immune cells have a prominent role in *Toxoplasma* infection and several studies indicate that A) the parasite can directly modulate the immune-system by injection of effector proteins and B) it uses immune-cells as Trojan horses to cross the blood brain barrier to reach the brain, where it encysts to reach chronic infection. The fact that mutants defective in iiEgress also have a deficiency in establishing a chronic infection fits the connection between rapid egress and *in vivo* propagation. Through the genomic analysis of several independent mutants with defects in iiDeath, iiEgress, and in some cases the capacity to establish a chronic infection in the brain, we show here that CDPK3 is key to calcium-dependent signaling and the virulence of the parasite *in vivo*.

## Results

### Sequencing of ionophore-induced egress mutant MBE1.1 reveals a missense mutation in the Calcium -Dependent Kinase 3

To determine the mutation responsible for the phenotypes observed with mutant strain MBE1.1, we performed a low coverage screen of its genome for candidate loci using 454 pyrosequencing. As a control we also sequenced the genome of RHΔ*hpt*, which is the strain used to generate the iiEgress mutants. Using the assembled genome of strain GT1 as a reference [22], we assembled both the RHΔ*hpt* and MBE1.1 genome sequences. Single nucleotide polymorphisms (SNP) between the parental and mutant strains were given a score from 0 to 1 based on the consistency with which individual reads of the mutant strain were different from that of the parental strain. We found a total of 451 SNPs with a score of .8 or higher between the parental strain and MBE1.1. Of these SNPs, 56 were within the transcribed region of predicted protein-coding genes but only 2 affected exons. The large number of SNPs in non-coding sequences between MBE1.1 and RHΔ*hpt*, may be due to the fact that the RHΔ*hpt* strain sequenced was maintained in culture for a prolonged period of time after the generation of MBE1.1 and likely drifted from the original parent strain.

**Figure 1:**
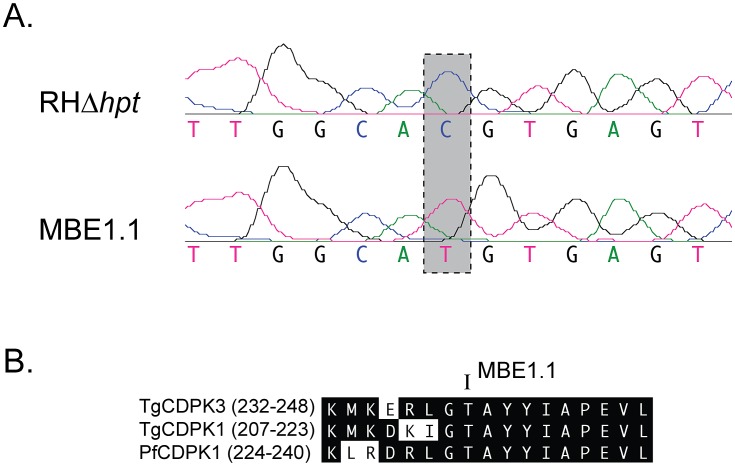
Mutation in *TgCDPK3* of strain MBE1.1. A. DNA sequence chromatogram of bases 4,684,206-18 of chromosome IX for both the parental (RHΔ*hpt*) and the iiEgress mutant (MBE1.1) strains are shown. Gray box highlights the SNP found in the mutant strain. B. A comparison of the equivalent of protein kinase superfamily subdomain VIII from TgCDPK3 (accession number ABA60892), TgCDPK1 (accession number XP_002371666) and PfCDPK1 (accession number XP_001349680) is shown. The Threonine (T) changed to an Isoleucine (I) in TgCDPK3 from the mutant strain MBE1.1 is indicated. The numbers of the amino acid shown are listed in parenthesis next to the name of each protein.

One of the mutations found in coding sequence (A428,372G in chromosome V, based on the ME49 genome) causes a silent mutation in gene *TgGT1_015610*. The other is a transition mutation of a C for T change in base 4,684,212 of chromosome IX, which causes a missense mutation in *TgGT1_041610*. This latter gene encodes a previously characterized calcium-dependent kinase, TgCDPK3 [23], [24]. Given that this was the only missense mutation we detected in our sequences, we confirmed the nucleotide change by sequencing a PCR fragment from genomic DNA, which included the potentially affected region. This experiment confirmed the C to T transition in MBE1.1 in relation to the parental strain (figure 1). This nucleotide mutation results in a change of threonine 239 for an isoleucine in TgCDPK3. This amino acid is within the catalytic domain of TgCDPK3 (see below, supplemental figure S1A) and it is conserved in 10 of the 11 CDPKs found in *T. gondii* (substituted for Ala in TgCDPK4A) and in all 7 CDPKs from *Plasmodium falciparum* (Supplemental figure S1B). Figure 1C shows a comparison of the region around Thr 239 with that of TgCDPK1 from *T. gondii* and of the closest homolog of TgCDPK3 in *Plasmodium falciparum*, PfCDPK1.

The mutation identified lies in the last base of exon 3 (supplemental figure S2A). Thus, the possibility of aberrant splicing as a result of the mutation was assessed by sequencing of cDNA and 3′ RACE (supplemental figure S2B), which both showed TgCDPK3 is expressed with the correct splicing. While we observe that the correctly spliced form is present in the mutant strain, it is possible that the level of TgCDPK3 transcript is affected by inefficient splicing. To explore this possibility, we performed the comparative Ct method of real-time PCR to assess *TgCDPK3* RNA levels in the parental and MBE1.1 strains. Using primers directed against alpha-tubulin as an endogenous control, we determined that the relative quantity of *TgCDPK3* RNA in MBE1.1 was 1.12±0.19 times that found in the parental strain, indicating that the *TgCDPK3* transcript is present at approximately wild-type levels in MBE1.1 (supplemental figure S3). These results show that the phenotypes observed with T239I are not due to effects of the mutation on the processing or stability of the TgCDPK3 transcript.

### TgCDPK3 complements the mutant phenotype of MBE1.1

**Figure 2:**
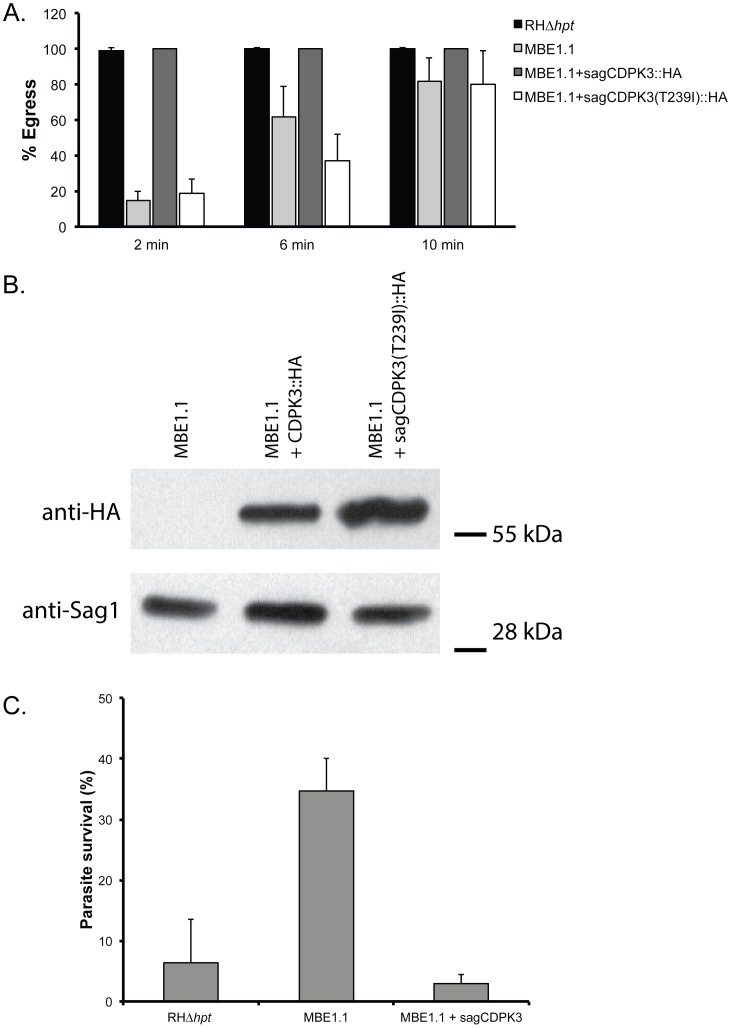
Phenotypic complementation of MBE1.1. A. Intracellular parasites of the parental (RHΔ*hpt*), mutant (MBE1.1), complemented (MBE1.1+sagCDPK3::HA) strains, as well as MBE1.1 parasites complemented with TgCDPK3 carrying the T239I mutation (MBE1.1+sagCDPK(T239I)::HA) were exposed to 1 µM A23187 for the time points indicated in the graph. Percentage egress represents the number of lysed vacuoles divided by the total number of vacuoles (lysed+intact). B. Exogenous copy of CDPK3::HA in the strains complemented with the wild type (MBE1.1+sagCDPK3::HA) or mutant gene (MBE1.1+sagCDPK3(T239I)::HA) was detected by western blot analysis using HA antibodies. Antibodies against the surface protein Sag1 were used to show equal loading. C. Extracellular parasites of the indicated strains were exposed to 1 µM A23187 for 45 minutes before being added to cells. Percentage survival was calculated by dividing the number of plaques formed by parasites treated with A23187 divided by the number of plaques formed by untreated parasites. For each treatment, all plaques in a well of a 12-well plate were counted. In B and C, each data point represents the average of four independent experiments and the error bars represent the standard deviation.

To determine whether the mutation identified within *TgCDPK3* is responsible for the delay in ionophore-induced egress, we introduced a C-terminally HA-tagged wild-type copy of the coding sequence under the control of the *T. gondii SAG1* promoter (sagCDPK3::HA), into MBE1.1. When this complemented strain is exposed to A23187 while intracellular it exhibits nearly wild-type levels of ionophore-induced egress (figure 2A): while only 14.8±5% of vacuoles of the MBE1.1 strain are lysed by 2 minutes, 100% of MBE1.1+sagCDPK3::HA vacuoles have been ruptured by the same time point (figure 2A). The level of egress in the complemented strain at 2 minutes was not statistically different (based on paired T test) than what is observed for the parental strain (99±1%). We observed the same complementation effect when the wild-type copy of *TgCDPK3* was under the control of a 2 kb region upstream of the *TgCDPK3* start codon (*i.e.* its own promoter, supplemental figure S4). Complementation with a TgCDPK3 allele carrying the T239I mutation of MBE1.1 did not complement the phenotype (figure 2A). To determine if the T239I mutation affected the overall expression of the exogenous protein, western blot analysis was performed on the strains complemented with the wild-type or the T239I strain. The results (figure 2B) showed approximately the same level of TgCDPK3 expression using the wild-type and mutant alleles.

Besides the delay in iiEgress, mutant MBE1.1 is also resistant to iiDeath. To investigate whether the T239I mutation in TgCDPK3 is also responsible for this phenotype we tested the extracellular sensitivity to calcium ionophore of the complemented strain (figure 2C). When extracellular parasites of either the parental, the mutant MBE1.1 or the wild type-complemented strain are treated with 1 µM A23187 for 45 minutes we observe significantly higher levels of survival in the mutant strain as compared to either the parental or complemented parasites (33.7% vs. 10.3% and 2.1%, respectively, significance determined by Anova).

### TgCDPK3 is localized to the parasite periphery in intracellular and extracellular parasites

**Figure 3:**
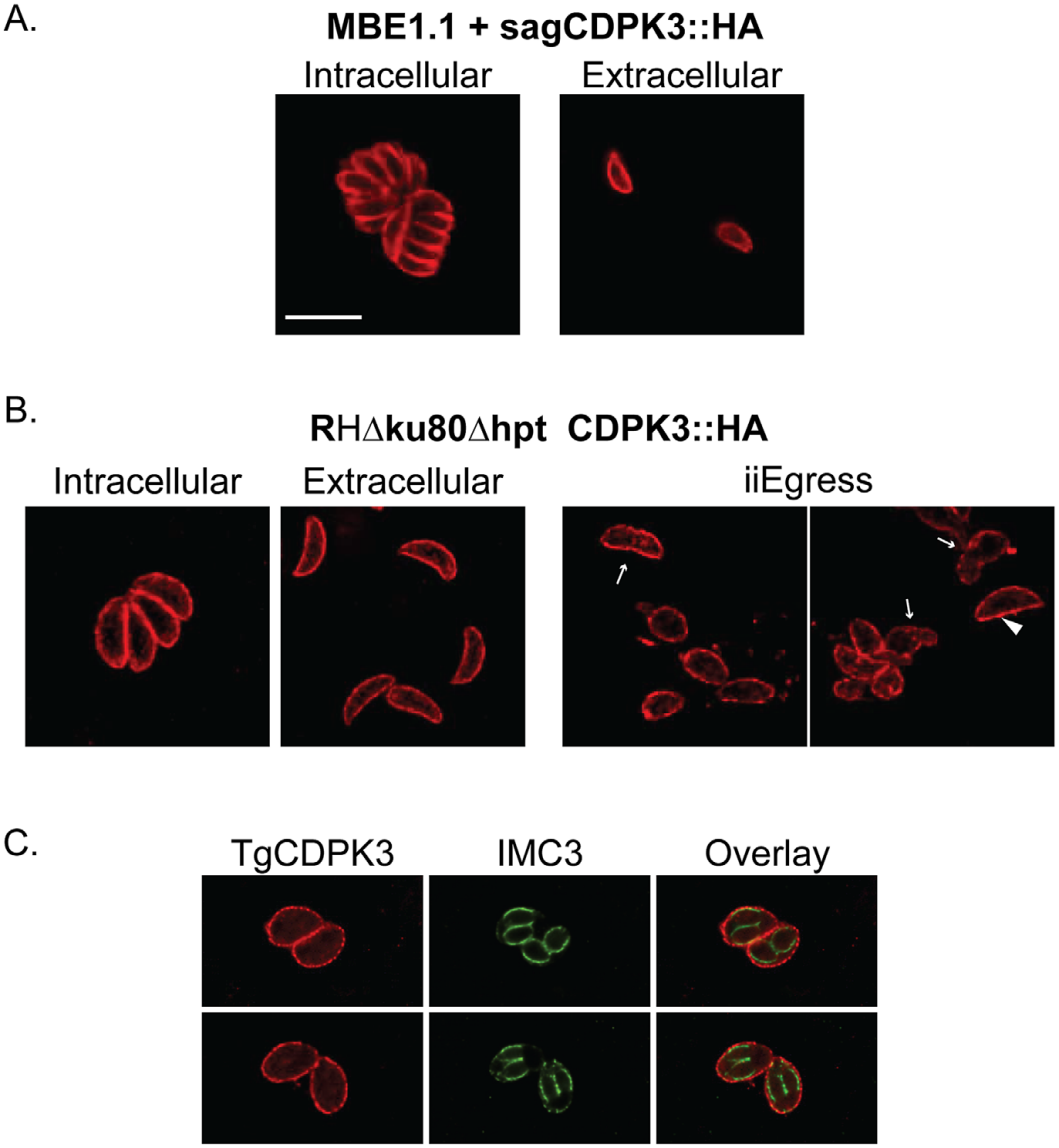
Localization of TgCDPK3. A. Intracellular and extracellular parasites of the MBE1.1 strain complemented with a wild-type copy of TgCDPK3 were stained with antibodies against the hemagglutinin (HA) tag present in the exogenous copy of TgCDPK3. B. Parasites with an HA tag in the endogenous copy of TgCDPK3 (RHΔ*ku80*Δ*hpt* CDPK3::HA) were stained with HA antibodies while intracellular and extracellular. In addition, intracellular parasites of the same strain were exposed to 1 µM A23187 for 2 minutes to induce iiEgress (two far right panels). In the iiEgress panels arrows point at parasites in the process of exiting or invading cells, arrow head points at a parasite that has remained inside of a host cell, while all other parasites have already completed egress. C. RHΔ*ku80*Δ*hpt* CDPK3::HA parasites were co-stained with HA antibodies and antibodies against the inner-membrane protein 3 (a gift of MJ Gubbels). Scale bar 10 µm.

As part of their molecular characterization of TgCDPK3, Sugi et al. [24] investigated the localization of the protein using antibodies generated in mice against a GST-CDPK3 fusion. They reported that in intracellular parasites TgCDPK3 is localized to the cytosol and partially to the apical end of the parasite, while in extracellular parasites the protein is located solely at the apical end. Interestingly, staining MBE1.1+CDPK3::HA parasites with HA antibodies showed that the transgenic protein was clearly localized to the periphery of both intracellular and extracellular parasites (figure 3A). In neither intracellular nor extracellular parasite did we observe distinct, apical localization.

To eliminate the possibility that this localization is the result of possible over-expression of the transgenic protein, we tagged the endogenous locus of TgCDPK3 with an HA tag in a parasite line lacking the KU80 gene (which decreases the rates of non-homologous recombination [25], [26]) to generate the parasite line RHΔ*KU80*Δ*hpt* CDPK3::HA. Immunofluorescence assays of this strain confirmed that the endogenous protein is in the periphery of the parasite regardless of whether the parasites are inside or outside human cells (figure 3B). To investigate whether this localization changed during calcium fluxes, we stained parasites that were induced to undergo egress with A23187 for 2 minutes. In these parasites the majority of the TgCDPK3 signal appeared to still be associated with the periphery of the parasite (figure 3B). This was the case even for parasites that were in the process of either exiting their host cell or invading a neighboring one, which can be recognized by the constriction around the parasite's body (arrows in figure 3B). Moreover, we observe that during parasite division, TgCDPK3 remains in the periphery of the mother cell and it is not present in the inner membrane complex (IMC), which is associated with the nascent parasites (figure 3C) and we detected with antibodies against IMC3 [27].

### Membrane localization is required for function of TgCDPK3 in iiEgress

**Figure 4:**
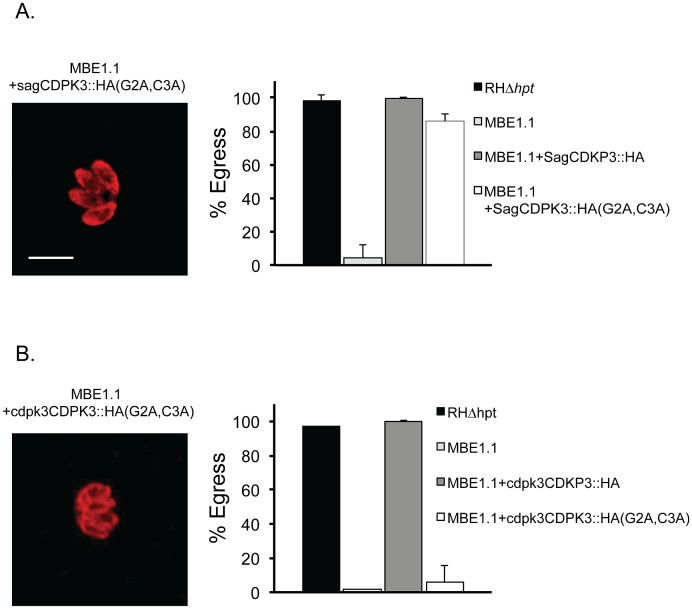
Effect of mutating myristoylation and palmitolation sites in TgCDPK3 localization and function. A construct with TgCDPK3 carrying mutations in amino acids 2 and 3 (TgCDPK3::HA(G2A,C3A)) expressed by either the Sag1 promoter (A) or the *TgCDPK3* promoter (B) was introduced into the MBE1.1 strain. Left panels show localization of TgCDPK3::HA(G2A,C3A) using HA antibodies. Right panels show percentage of parasites of the strain indicated that undergo egress after 2 minutes exposure to 1 µM A23187. Each data point represents the average of four independent experiments and the error bars represent the standard deviation. Scale bar is 10 µm.

To further validate the localization of TgCDPK3::HA, we mutated the two amino acids in the N terminus of the protein that are predicted to be modified post-translationally: the glycine at position 2 is predicted to be myristoylated (Myristoylator) [28] and the adjacent cysteine at position 3 is predicted to be palmitoylated (CSS-Palm 2.0) [29]. To determine whether localization to the membrane is dependent on these amino acids, we replaced them both for alanine in the rescue vector and introduced this into the MBE1.1 mutant and assessed both localization and function. In parasites expressing TgCDPK3(G2A, C3A) containing an HA tag, we detect the protein throughout the cytoplasm with little if any specific association with the parasites' plasma membrane (figure 4A). This suggests that modification at the N terminus of the protein is likely required for proper localization of TgCDPK3. Surprisingly, however, we observed that TgCDPK3(G2A,C3A)::HA was able to completely rescue the iiEgress phenotype of MBE1.1. While this might indicate that localization is not critical for the function of TgCDPK3 in induced egress, it is also possible that when over-expressed (as occurs with the strong SAG1 promoter, supplemental figure S4C), enrichment in the periphery is not necessary to complement the mutant phenotype. To address this possibility, we expressed TgCDPK3(G2A,C3A) under its own promoter in the MBE1.1 strain. While the wild-type TgCDPK3 expressed off its own promoter complemented the iiEgress phenotype of MBE1.1, this was not the case with the TgCDPK3(G2A,C3A) allele (figure 4B). This indicates that, under normal expression conditions, localization of TgCDPK3 to the membrane is needed for its function in iiEgress.

### Three additional, independently isolated iiEgress mutants also carry mutations in CDPK3

**Figure 5:**
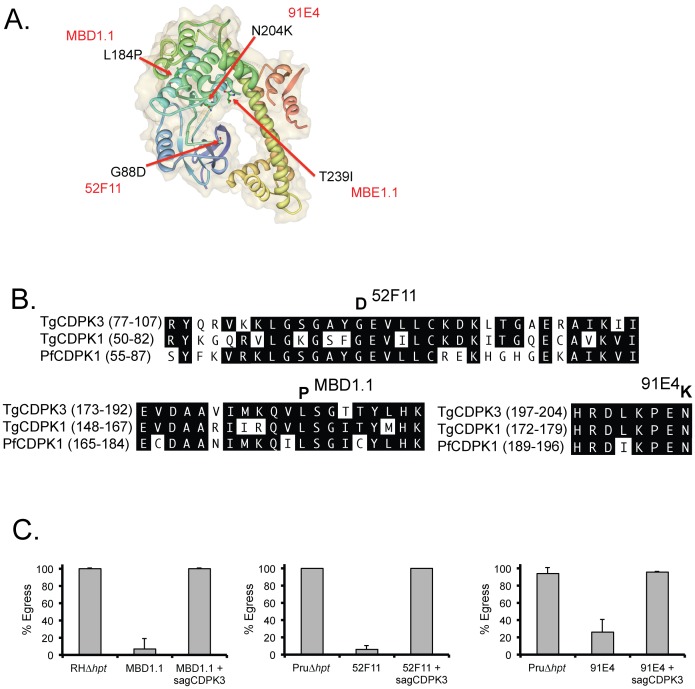
Mutations in TgCDPK3 in mutant strains MBD1.1, 52F11 and 91E4. A. The positions of the amino acids mutated in each of the four iiEgress mutants are shown within the TgCDPK3 structure. Schematic was rendered in Protein Workshop using the published structure of TgCDPK3 bound to the ATP analog 5-[(Z)-(5-chloro-1, 2-dihydro-2-oxo-3H-indol-3- ylidene)methyl]-N-[2-(diethylamino)ethyl]-2, 4-dimethyl-1H-pyrrole-3-carboxamide (accession 3HZT). B. The protein sequences show the amino acids in TgCDPK3 surrounding the glycine (G) to aspartic acid (D) change in 52F11 (top), those around the leucine (L) to proline (P) change in MBD1.1 (bottom left) and the HRD catalytic domain of TgCDPK3 with the Asparagine mutated in 91E4 (bottom right), all compared to the equivalent regions in homologs TgCDPK1 and PfCDPK1. C. Intracellular parasites of the parental (RHΔ*hpt* for MBD1.1, PruΔ*hpt* for 52F11 and 91E4), mutants (MBE1.1, 52F11 and 91E4) and the strains complemented with the wild-type copy of TgCDPK3 were exposed to 1 µM A23187 for 2 minutes. Percentage egress represents the number of lysed vacuoles divided by the total number of vacuoles (lysed+intact). Each data point represents the average of four independent experiments and the error bars represent the standard deviation.

Besides MBE1.1, the original selection for mutants with a delay in iiEgress also resulted in the isolation of MBE3.3, which unlike MBE1.1 does not have a resistance to iiDeath (Table 1). Selecting directly for resistance to ionophore-induced death resulted in the isolation of MBD1.1, which also has a delay in iiEgress, and MBD2.1, which has normal induced egress (Table 1) [17]. We sequenced the *TgCDPK3* locus in all of these strains and whereas MBE3.3 and MDE2.1 both harbor a WT CDPK3 sequence (data not shown), MBD1.1 carries a mutation in CDPK3, resulting in a Leu to Pro conversion in amino acid 184 (figures 5 A and D and supplemental figure S5), which lies within a helical region [30]. Introduction of a wild-type copy of TgCDPK3 under the *SAG1* promoter complements the iiEgress phenotype of MBD1.1 (figure 5E). Thus, of the 4 mutants isolated by Black *et al.* only those that have both iiEgress and iiDeath phenotypes carry mutations in TgCDPK3, which strongly points towards this protein being essential in these processes.

As externally triggered egress has been suggested to be important for dissemination of the parasites, we previously screened a panel of chemically mutagenized, signature-tagged mutants that had decreased virulence in mice and identified two, 52F11 and 91E4, that also to have a defect in iiEgress and iiDeath [18]. We sequenced the *TgCDPK3* locus of both and identified a mutation in TgCDPK3 in each (figures 5B and C and supplemental figure S5). In 52F11 we detected a G for A change, which results in a Gly for Asp change in amino acid 88 of TgCDPK3 (figures 5B, 5D and S1 and table 1). Sequencing of TgCDPK3 in the 91E4 mutant shows a C for G, which causes an Asn for Lys mutation in amino acid 204 of TgCDPK3 (figures 5C, 5D and S1 and table 1). Furthermore, the iiEgress phenotype in both of these mutants is complemented by the wild-type copy of TgCDPK3 (figure 5E). The fact that both 52F11 and 91E4 have mutations in TgCDPK3 is highly significant, as they both have a significant reduction in the number of cysts formed during mice infections [18]. Thus, TgCDPK3 appears to play an important role *in vivo*.

### Mutations in TgCDPK3 that result in egress phenotypes drastically reduce CDPK3 function

**Figure 6:**
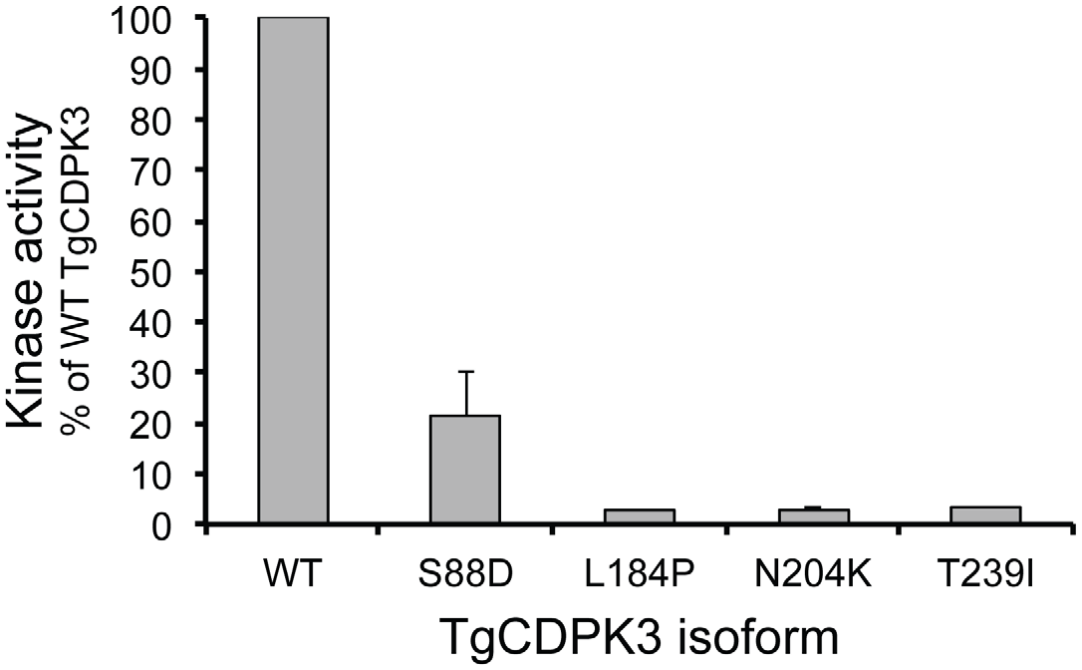
*In vitro* kinase activity of wild-type and mutant TgCDPK3. Recombinant wild-type (WT) or mutant (G88D, L184P, N204K and T239I) TgCDPK3 were tested for activity against syntide-2 in the presence of CaCl_2_. Whereas wild-type TgCDPK3 shows robust activity against the substrate, mutagenesis of G88D, L184P, N204K or T239I inactivates kinase activity. Error bars represent standard deviation from three independent experiments.

Analysis of the published structure of TgCDPK3 [30] revealed that all identified mutation sites (G88, L184, N204 and T239) lie within the kinase domain of CDPK3 and are predicted to inactivate kinase activity (figure 5A). Glycine 88 (mutated to Asp in 52F11) is part of the almost universally conserved Gly-X-Gly-X-X-Gly-X-Val domain, which anchors the non-transferable phosphates of ATP [31]. Leucine 184 (mutated in MBD1.1 to Pro) is within an alpha helix [30], [32], which would likely be disrupted by the introduction of a proline. Asparagine 204, which is affected in 91E4, is conserved in virtually all kinases and is part of the HRD catalytic domain of this class of protein kinases (HRDLKxxN) [31], [33]. Threonine 239, which is mutated in MBE1.1 is located in the kinase activation loop [31] which plays an important role in the recognition of peptide substrates [31]. The equivalent amino acid in PfCDPK1 is also a threonine and it has been shown to be auto-phosphorylated [34]. To test if all identified mutations inactivate TgCDPK3 we expressed wild-type TgCDPK3 and versions carrying the mutations found in MBE1.1, MBD1.1 and 52F11 and 94E1 (T239I, L184P, G88D and N204K, respectively) in bacteria. We compared kinase activity against the short peptide, syntide-2, which was previously used to monitor TgCDPK3 activity [32]. Whereas wild-type TgCDPK3 showed high levels of phosphorylation of the substrate, introduction of any of the mutations found in the iiEgress mutants reduced TgCDPK activity to at most 20% of wild-type levels (figure 6).

### The PfCDPK1 inhibitor purfalcamine inhibits iiEgress in *T. gondii*

**Figure 7:**
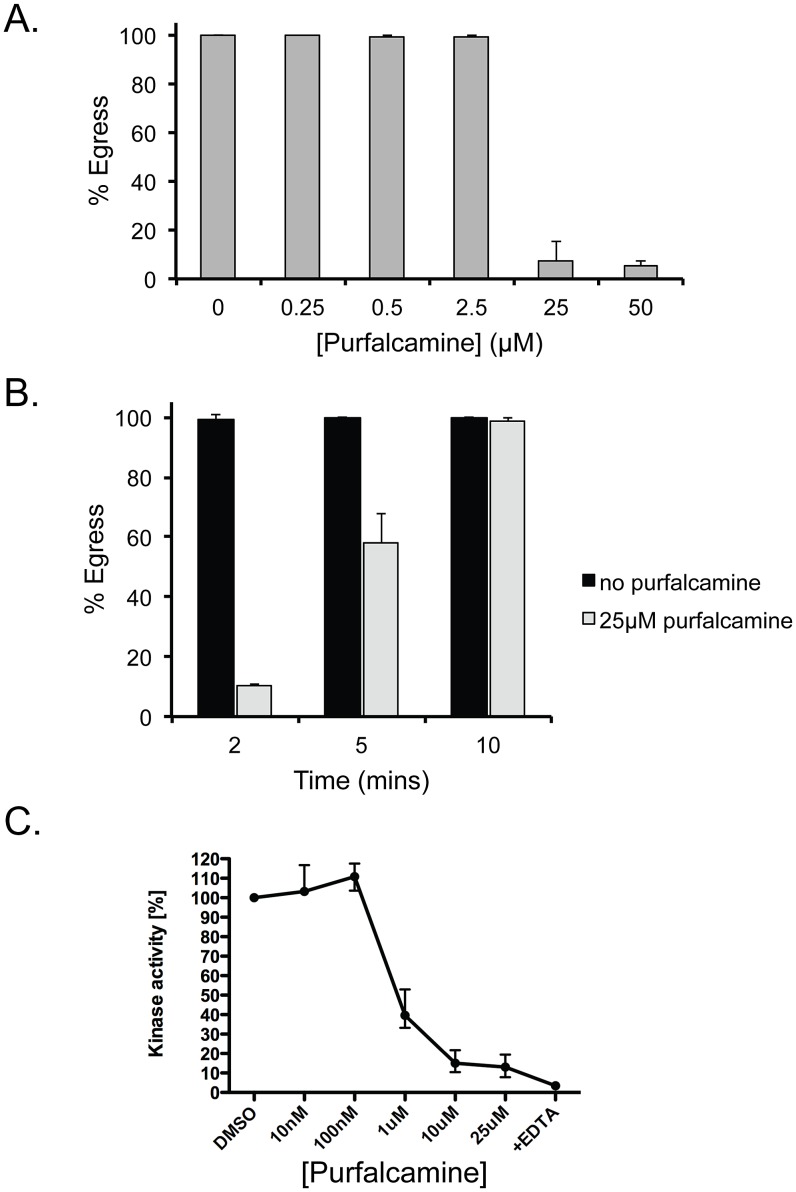
Effect of purfalcamine on iiEgress and TgCDPK3 activity. A. Intracellular parasites were pre-treated with the indicated amount of purfalcamine for 15 minutes before being exposed to 1 µM A23187 for 3 minutes to induce egress. B. Parasites were pre-incubated with 25 µM A23187 for 15 minutes before treatment with 1 µM A23187 for the time indicated in the X-axis. Percentage egress represents the number of lysed vacuoles divided by the total number of vacuoles (lysed+intact). Each data point represents the average of four independent experiments and the error bars represent the standard deviation. C. The effect of purfalcamine on TgCDPK3 activity was tested in *in vitro* kinase assays. Each data point indicates the mean of 4 experiments; error bars represent the standard deviation.

A screen for inhibitors of recombinant PfCDPK1, the closest homolog of TgCDPK3 in *Plasmodium falciparum*, identified the 2,6,9-trisubstituted purine purfalcamine, which was shown to have anti-plasmodial activity in culture experiments [35]. PfCDPK1 was confirmed as the target of purfalcamine through affinity chromatography of parasite lysate [35]. In addition, although at higher concentrations than what is needed to inhibit PfCDPK1, purfalcamine was shown to render *T. gondii* unable to invade [35]. Consequently, we tested whether purfalcamine could affect iiEgress, which we have now shown to be TgCDPK3 dependent. Accordingly, we pre-treated intracellular parasites with different concentrations of purfalcamine and then exposed them to 1 µM A23187 for 3 minutes. We noted that at both 25 µM and 50 µM purfalcamine largely blocked iiEgress 2 minutes after addition of the ionophore (figure 7A). These concentrations are identical to the ones found to have anti-Toxoplasma activity by Kato et al. [35]. To further analyze the inhibition of iiEgress by purfalcamine we treated intracellular parasites with 25 µM purfalcamine for 15 minutes before exposing them to 1 µM A23187 for 2, 5 and 10 minutes (figure 7B). While 100% of untreated parasites undergo egress by 2 minutes, we measured 10.5±0.5%, 57.8±9.7% and 98.7±1.4% egress at 2, 5 and 10 minutes of ionophore exposure respectively when parasites were pre-treated with purfalcamine. Thus, purfalcamine causes a delay in iiEgress that is very similar to what is seen when TgCDPK3 is mutated (figure 2B) suggesting that it could be a target of purfalcamine in *T. gondii*.

To determine whether purfalcamine can inhibit TgCDPK3 directly, we tested its effect on recombinant TgCDPK3. As shown in figure 7C, purfalcamine specifically inhibits CDPK3 activity against syntide-2 (IC50 = 800 nM); however, even at high concentrations (25 uM) it does not completely inactivate the kinase as the EDTA control shows (the EDTA control chelates the calcium in the reaction which is required for CDPK3 activity).

### A TgCDPK3 knockout shows that it is not essential but has a minor influence on the rate of division of *T. gondii*

**Figure 8:**
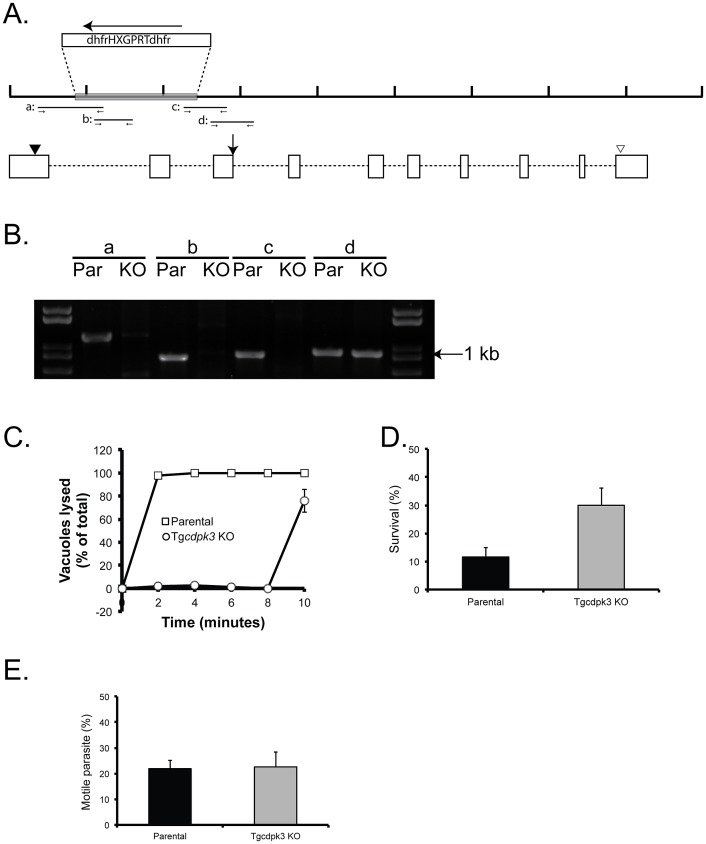
Establishment and analysis of a *Tgcdpk3* knockout strain. A. Schematic diagram of the TgCDK3 genomic locus and the region disrupted in the knockout strain. The top black line represents the genomic DNA region with each tick being 1,000 bases. The gray box represents the region of the TgCDPK3 locus that is replaced by the *hpt* selectable marker (dhfrHXGPRTdhfr), which is transcribed in the opposite direction from TgCDPK3 (as indicated by arrow pointing left). The relative positions of exons (white boxes) and introns (dashed lines) are shown below. Black arrowhead indicates position of start codon while the white arrowhead points at the stop codon. Lines labeled a, b, c and d represent the regions amplified by PCR to test for the disruption of *TgCDPK3*. B. PCR products of the parental strain, RHΔ*hpt*Δku80, and the TgCDPK3 KO strain, RHΔ*hpt*Δ*ku80*Δ*cdpk3*+HPT are shown. Labels above lanes indicate the DNA fragment amplified. Fragments a, b and c cannot be amplified if the T*gCDPK3* locus has been disrupted by the *hpt* marker. Fragment d is expanded from both the parental and knockout strain and it serves as a control. C. Intracellular parasites of either the parental or knockout strain were treated with 1 µM A23187 for the time indicated. Percentage egress represents the number of lysed vacuoles divided by the total number of vacuoles (lysed+intact). Each data point represents the average of four independent experiments and the error bars represent the standard deviation. D. Extracellular parasites of either the parental or the knockout strain were exposed to 1 µM A23187 for 45 minutes before being added to cells. Percentage survival was calculated by dividing the number of plaques formed by parasites treated with A23187 divided by the number of plaques formed by untreated parasites. Each data point represents the average of three independent experiments and the error bars represent the standard deviation. E. Parasites were allowed to settle on glass in intracellular buffer, which was changed to extracellular buffer to initiate motility. After 15 minutes parasites were fixed and the percentage of parasites that had moved was determined by counting parasites that were associated with trails of surface proteins left behind during gliding. Data is from 3 independent studies and the error bars are the standard deviation.

To assess the impact of a complete loss of TgCDPK3 we generated a knockout strain of *TgCDPK3* and analyzed its phenotype. For this purpose we made a construct, pKOCDPK3, which consists of the selectable marker hypoxanthine-xanthine-guanine-phosphoribosyltransferase (*HPT*) flanked by fragments of *TgCDPK3*, such that when it integrates into the genome by double homologous recombination it replaces a 1543 bases fragment from the *TgCDPK3* locus with *HPT* (figure 8A). This event would eliminate an entire exon and introduce a complete gene, including a polyA addition site, driven off the opposite strand, into its middle. This should result in no functional TgCDPK3 protein being produced. Following transfection of a parental RHΔ*hpt* strain of *T. gondii* with pKOCDPK3 we established stable *HPT*-positive clones, which were tested by PCR to determine if they harbored a disruption of *TgCDPK3*. Unfortunately, six independent attempts failed at producing a knockout strain, which suggested that TgCDPK3 might be essential for parasite survival. The recent creation of a parasite line, RHΔ*hpt*Δ*ku80* [25], [26], that is deficient in non-homologous end-joining, allowed us to revisit the disruption of TgCDPK3. Using this strain and the same approach described above, we were able to establish a parasite clone, RHΔ*hpt*Δ*ku80*Δ*cdpk3*+HPT (Tg*cdpk3* KO), that was confirmed to be disrupted at the TgCDPK3 locus by 3 independent PCR reactions each with different primer sets (figure 8B).

Using the knockout strain we first tested if it recapitulated the iiEgress and iiDeath phenotypes of the TgCDPK3 missense mutants. The knockout strain exhibited a marked delay in iiEgress defect akin to the one seen with the TgCDPK3 missense mutants: while the parental strain exhibited 98.0±1.3% egress at 2 minutes, at the same time point only 1.6±2.9% of knockout strain vacuoles had lysed (figure 8C). This phenotype was also seen with a second independent knockout clone (data not shown). Just as with the TgCDPK3 point mutants, the knockout strain was also resistant to the lethal effects of extracellular exposure to the calcium ionophore (figure 8D). Given previous reports that recombinant TgCDPK3 could phosphorylate components of the glideosome motility system in vitro [24] we tested whether the knockout strain for defects in this process. We allowed parasites to settle onto glass cover slips in a buffer mimicking intracellular environment (IC buffer), which keeps the parasites in a pseudo-intracellular, non-motile state. This buffer was changed to an extracellular buffer (EC buffer) to allow initiation of motility, and after 15 minutes the parasites were fixed and stained for the surface marker Sag1, which allows us to detect the parasites as well as the trails they leave behind during gliding. We did not notice any difference in the percentage of parasites that were associated with trails between the parental and the Tgcdpk3 knockout strains (figure 8E). Furthermore, we did not detect any obvious difference in either the shape or length of the trails (data not shown), indicating that parasite motility is not affected by the disruption of TgCDPK3.

**Figure 9:**
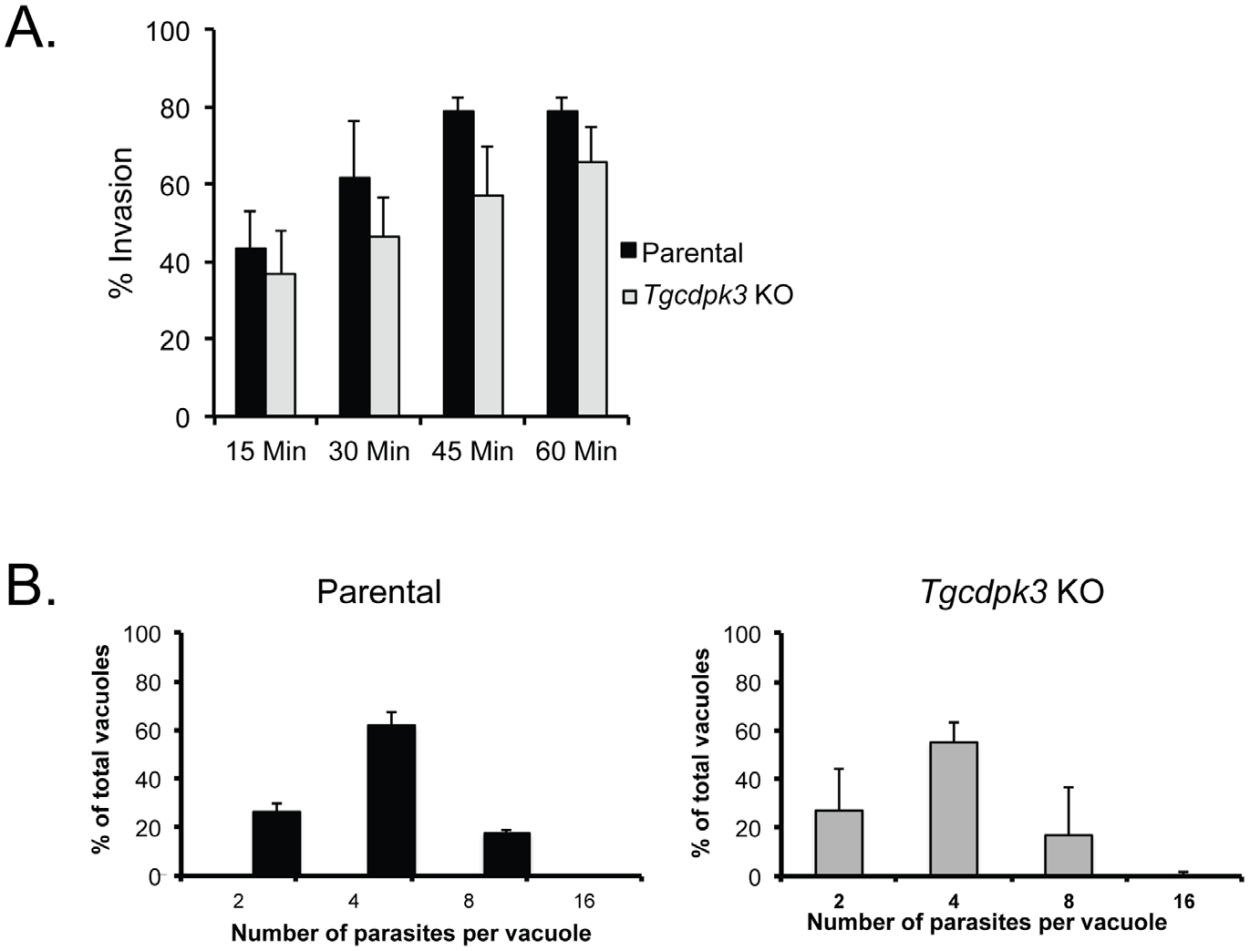
Propagation related phenotypes of TgCDPK3 knockout strain. A. Parental or knockout parasites were allowed to invade confluent HFFs for the amount of time indicated. Cultures were then fix and the percentage of parasites that were inside of the cells were determine for each data point. The data represents the average of 3 independent assays and the error bars represent the standard deviation. B. Parasites were allowed to divide for 24 hours and a total of at least 100 vacuoles were inspected to determine the number of vacuoles with 2, 4, 8 or 16 parasites within. Data are the average of 3 independent experiments and the error bars are the standard deviations.

The fact that we were not able to obtain a knockout strain of TgCDPK3 in a standard (KU80-expressing) parental strain could be due to the knockout strain being outgrown by others that incorporate the selectable marker without completely deleting the *TgCDPK3* locus. Using the Δ*ku80* strain allows for enrichment of parasites with insertion of the selectable marker by homologous recombination and reduces competition with wild-type parasites. Based on the observation that parasites of the knockout strain appear to lyse a fibroblast monolayer at a slower rate than the parental counterpart we suspected that it had a defect on one or more of the steps that influence propagation: invasion, division, and normal egress. When we inspected vacuoles of the knockout strain between 36 and 60 hours post invasion (period of time when egress starts occurring) we detected none with an abnormally high number of parasites, indicating that the knockout strain does not have a delay in normal egress (data not shown). To investigate if there is a delay in invasion, we allowed parasites to invade cells for between 15 and 60 minutes and observed no difference in the percentage of parasites that invade cells between the parental and knockout strain (figure 9A).

To determine whether the knockout strain divides at a different rate from that of the parental strain we allowed parasites from each strain to invade cells for 4 hours and then after a further 20 hours we counted the number of parasites per vacuole for at least 100 vacuoles per sample. The results showed a markedly different distribution of vacuole sizes between the two strains: while the majority of parental strain vacuoles, 79.3±7.3%, had 8 of more parasites, 82.4±25.4% of Tg*cdpk3* knockout vacuoles had 4 or fewer parasites per vacuole (figure 9B). This indicates that the knockout strain divides at a slower rate, which likely accounts for the overall slower propagation observed.

## Discussion

Pathogenic intracellular parasites, including *Plasmodium spp.*, the causative agent of malaria and *T. gondii* rely on calcium-signaling and kinases to elicit specific cellular responses and to interact with their hosts. They differ from their mammalian hosts, however, in having a family of calcium-dependent protein kinases (CDPKs). These unique kinases are abundant in plants (*Arabidopsis thaliana* has 42 CDPKs [36]), ciliates, and parasites of the phylum Apicomplexa, but are absent from animal cells. Consequently, parasite CDPKs have been suggested and studied as potential targets of drug therapies. With this in mind significant effort is being put forward to characterize the function of the many CDPKs found in *T. gondii* and *P. falciparum* [37]. Using a forward genetic approach, we have determined that TgCDPK3 is key to the parasite's response to calcium fluxes in *T. gondii* and shown that the virulence of strains harboring a mutation in this gene is substantially reduced in a mouse model [18].

The function of TgCDPK3 within the life cycle of *T. gondii* had not previously been elucidated although *in vitro* experiments using recombinant protein have shown that TgCDPK3 is capable of phosphorylating *T. gondii* Aldolase 1, an important component of the gliding motility machinery of this parasite [24]. While a role for TgCDPK3 in modifying components of the so-called glideosome is consistent with the fact that motility is Ca^2+^-dependent and our results that TgCDPK3 localizes to the periphery of the parasite, mutants in *TgCDPK3* show no observable defect in motility [17]. Thus, the biological relevance of *in vitro* Aldolase phosphorylation, at least as a function of motility, is unclear.

Interestingly, the closest homolog of TgCDPK3 in *Plasmodium falciparum*, PfCDPK1, is also localized to the periphery of the parasite in its merozoite stage [38], [39] and recombinant protein can phosphorylate glideosome components in *in vitro* tests [38]. Specifically, recombinant PfCDPK1 can phosphorylate both the myosin light chain (MTIP) and the glideosome-associated protein GAP45 [38]. Both of these proteins are phosphorylated in the parasite [38], [40] and one of the GAP45 sites phosphorylated *in vitro* was shown to also be phosphorylated *in vivo* suggesting that PfCDPK1 might be responsible for this phosphorylation within the parasite. This modification of GAP45 could indicate a role in assembly of the glideosome since it has been shown that the phosphorylation state of GAP45 regulates assembly of the motor complex in *T. gondii* [41]. However, that fact that we do not observe a measurable phenotype in gliding motility in *TgCDPK3* mutant parasites suggests that, at least in *T. gondii*, a role for TgCDPK3 in motility *per se* is unlikely.

While it is tempting to speculate that TgCDPK3 and PfCDPK1 directly regulate the function or assembly of the motility apparatus given their localization, other mechanisms by which these proteins might influence Ca^2+^-dependent motility should be considered. Motility in both *T. gondii* and *Plasmodium* depends on highly regulated fluxes of calcium and, prior to that, sensing of potassium levels, as only low levels of potassium allow motility, egress or invasion [42]–[44]. Anything that disturbs this ion homeostasis would likely affect events such as motility, invasion and egress. This is underscored by the observation that a knockout of a sodium/hydrogen exchanger, *TgNHE1*, results in a dysregulation of Ca^2+^ homeostasis and a delay in iiEgress [19]. Several plant CDPKs have been implicated in the regulation of ion homeostasis by phosphorylating proteins such as inward-rectifying K+ channels [45], [46], Ca^2+^ ATPases [47] and H^+^ pumps [48]. Thus, it is plausible that, upon activation by calcium, TgCDPK3 controls the amplitude and duration of the calcium fluxes by phosphorylating ion channels and transporters.

While a reduction of the calcium fluxes induced by the ionophore would delay egress, it could also allow extracellular parasites to resist the detrimental effects of exposure to the ionophore. Thus, effects on the amplitude or duration of calcium fluxes could be a factor connecting the iiEgress and iiDeath phenotypes that we observed. Defects in ionic homeostasis could also easily explain the delayed division of the knockout strain, as many steps of division and cytokinesis are calcium-dependent. Nonetheless, there are many other potential connections between all the phenotypes observed, including energetics, motility and secretion. Alternatively, TgCDPK3 could have multiple substrates and the phenotypes observed in the mutants could be due to parallel rather than overlapping pathways with only the kinase in common. Regardless, a better understanding of the mechanisms connecting the phenotypes requires the identification of the TgCDPK3 targets.

The main phenotype of our TgCDPK3 mutant strains when grown in culture is an inability to respond to artificially induced calcium fluxes. Careful phenotypic analysis of these mutants did not reveal any delay in normal egress parasite division, motility or micronemal secretion, *in vitro*, although a slight defect in invasion was detected [17]. This contrasts with results using purfalcamine, a potent and reportedly specific inhibitor of PfCDPK1, which completely inhibited the ability of extracellular *T. gondii* parasites to invade cells, leading to the conclusion that TgCDPK3 is likely to be essential for invasion [35]. Given our results, it seems likely that in *T. gondii*, purfalcamine has other targets besides TgCDPK3, as we do not observe a strong invasion phenotype in parasites lacking CDPK3 activity. It is interesting to note that we observed inhibition also of recombinant TgCDPK1 (data not shown), which has been associated with regulating invasion in *Toxoplasma gondii*. But whatever the other targets of purfalcamine are, we were able to show that purfalcamine inhibits TgCDPK3 and that iiEgress is affected in a manner similar to what is seen when TgCDPK3 is mutated.

The presence of two independent knockout lines proves the nonessential role that TgCDPK3 plays in the lytic cycle in cell culture. However, complete loss of TgCDPK3 function also results in a replication phenotype that was not observed in the mutant parasite lines. Whether this is a result of complete absence of TgCDPK3 activity (we cannot exclude minimal residual kinase activity in the mutants *in vivo*) or genetic manipulation of the locus remains unclear at the time. But whatever the cause for the growth phenotype might be, our data suggest that parasites lacking TgCDPK3 have an egress phenotype undistinguishable from the mutants.

A reason why in tissue culture our mutants only exhibit egress phenotypes during artificial induction is likely due to the fact the iiEgress phenotype represents a delay of only about 10 minutes which is not measurable in the normal 48–72 hour lytic cycle *in vitro* [49]. The function of TgCDPK3 will be critical in settings in which the parasite is required to rapidly exit a host cell. One such context is during the acute stages of an *in vivo* infection. Tomita *et al.* showed that in peritoneally infected mice, most *T. gondii* parasites divide at most 2 times before being induced by inflammatory cells to undergo egress in a calcium-dependent manner [21]. A possible mechanism for this *in vivo* rapid egress is suggested by the work of Persson *et al.* in which they observed that either death-receptor ligation or perforin exposure can trigger egress from infected T cells through the induction of intracellular calcium fluxes [20]. Consistent with this observation, it has been shown that after intraperitoneal inoculation of *T. gondii* into mice, parasites “jump” from infected dendritic cells (DC) to natural killer (NK) cells by a process that includes perforin-dependent killing of the infected cell and calcium-dependent egress from the dying cell [20]. Such cell jumping has also been observed *in vivo* [50]. Whether such a phenomenon is related to the decreased virulence of the 52F11 and 94E1 mutants [18] will require detailed *in vivo* characterization of these and complemented mutants. The results presented here, however, make clear that TgCDPK3 is a pivotal protein in the parasite's life *in vitro* and *in vivo*.

## Materials and Methods

### Parasite and host cell maintenance and reagents

RH strain parasites lacking a functional *hpt* gene, RHΔ*hpt* [51] and mutant parasites MBE1.1, MBE3.3, MBD1.1, MBD2.1, 52F11 and 91E4 [17] were maintained by passage through human foreskin fibroblasts (HFFs, culture cells obtained from ATCC) at 37°C and 5% CO_2_. Normal culture medium was Dubelcco's Modified Eagle Medium (DMEM) supplemented with 10% FBS, 2 mM L-glutamine and 100 units penicillin/100 µg streptomycin per ml. Ionophore assays were performed using Hanks Balanced Salts Solution (HBSS) supplemented with 1 mM MgCl_2_, 1 mM CaCl_2_, 10 mM NaHCO_3_, 20 mM Hepes, pH 7.2 (HBSSc). The calcium ionophore A23187 (Sigma) was dissolved in DMSO at 1 mM to make a stock solution.

### Genome sequencing

Extracellular parasites from strains MBE1.1 and RHΔ*hpt* (the parental strain) were purified through a 3 microns filter to eliminate human cell contamination. Genomic DNA from both strains was isolated using the DNeasy Blood and Tissue Kit (Qiagen). The DNA was then processed for emulsion PCR and sequenced on the Roche 454 GS FLX titanium platform. Sequencing resulted in a total of 674,003 reads (228,308,310 bases). Adapter sequences were removed from the raw reads using cross_match v1.08, a part of the Consed software package [52], and regions of poor quality (based on PHRED score) excluded using Lucy [53] with parameters: minimum good_sequence_length = 50, max_avg_error = 0.002, and max_error_at_ends = 0.002. Reads were further filtered using BLAT [54] to map the reads to the reference genome, and reads that matched over less than 70% of their length, or had 4 or more inserts were excluded [54] to map the reads to the reference genome, and reads that matched over less than 70% of their length, or had 4 or more inserts were excluded. After filtering, 147,903 sequence reads (51,249,990 bases) remained for the MBE strain, and 170,239 reads (60,381,739 bases) for the RH strain. This set of 318,142 filtered reads was then mapped to the reference genome using the Roche gsMapper software, where 316,992 (99.13%) of the reads were successfully mapped. The resulting differences file was parsed using a newly developed Java application, PyroSNP, to identify potential SNPs. The PyroSNP program parses the individual blocks (loci) in the 454AllDiffs file and identifies potential SNPs between the two mapped strains. Each potential SNP detected is assigned a “SNP score” ranging from 0 to 1 where a score of 0 indicates that a polymorphism occurs in the reads for each strain equally, and a score of 1 indicates that all the reads for each strain have a unique polymorphism. The specific algorithm is detailed in the PyroSNP documentation. Thus, the SNP score quantifies how consistently different samples are at this particular locus. Additionally, the PyroSNP application incorporates read quality information from the Roche454 output, allowing users to distinguish SNPs of low quality. The output from the PyroSNP parser application was then visualized using the SNPviewer application also developed to aid in SNP detection through complex sorting and the ability to view a potential SNP in its genetic context. The PyroSNP package is available at: (http://www.uidaho.edu/research/ibest/Tools).

Mutation in MBE1.1 was confirmed by sequencing a fragment of genomic DNA from both the parental and mutant strains obtained by PCR. The fragment was amplified using primers 5′ gcgcgttctcaggatgttcgt 3′ and 5′ cagtgtatctgcaacaaccaga 3′ and encompassed bases 4,683,601 to 4,684,889 of chromosome IX.

### Sequencing of TgCDPK3 genomic locus and transcript

To obtain the sequence of the TgCDPK3 transcript, total RNA from RHΔhpt and MBE1.1 parasites was purified using RNeasy purification kit (Qiagen) and reverse transcribed using an oligo dT primer and the SuperScript III First Strand Synthesis System (Invitrogen). The resulting cDNA was used as a template for a PCR reaction using primers 5′-gaggaggcgagttgttgac-3′ and 5′-ggagcagaacttgacgatcc-3′. The resulting fragment was cloned into pCR2.1-TOPO TA vector (Invitrogen). Five different clones were sequenced using T7 and M13reverse.

For 3′ RACE, cDNA from either RHΔ*hpt* or MBE1.1 parasites was used as a template in nested PCR reactions using the GeneRacer (Invitrogen) 3′ RACE primers in combination with 5′-gaggaggcgagttgttgac-3′ (for the primary reaction) and 5′-gctctctggcaccacttacc-3′ (for the nested reaction). The resulting fragment was cloned into pCR2.1-TOPO TA vector (Invitrogen). Five different clones were sequenced using T7 and M13reverse.

To sequence the TgCDPK3 locus in the different iiEgress mutants genomic DNA was obtained with DNeasy kit (Qiagen). PCR fragments were obtained using 9 different primer pairs as to span the entire genomic region (Supplemental figure S6) and sequenced directly.

### Real-time PCR

Total RNA was isolated from intracellular parasites using the RNeasy Plus Mini Kit (Qiagen). The RNA was reverse transcribed using an oligo dT primer and the SuperScript III First Strand Synthesis System (Invitrogen). The cDNA was used for Comparative Ct real-time PCR. Briefly, the cDNA was used in conjunction with 2x SYBR Green Master Mix (Applied Biosystems) and primer pairs directed against CDPK3 (5′-CTTGTCATGGAGGTGTACCG-3′ and 5′-GTAAGTGGTGCCAGAGAGCA-3′) or alpha tubulin (5′-ACGCCTGCTGGGAGCTCT-3′ and 5′-TCGTCACCACCTCCAATGG-3′), which was used for normalization. Real time PCR was carried out using the StepOne Plus Real Time PCR system (Applied Biosystems).

### Quantification of ionophore-induced egress

The efficiency of egress after calcium ionophore exposure was determined using established protocols [18]. In brief, parasites were added to each well of a 24-well tissue culture plate containing confluent HFFs at a multiplicity of infection (MOI) of 1. After 30 hours of growth, the parasites were incubated at 37°C in HBSS containing 1 µM A23187 calcium ionophore for time periods ranging from 0 to 10 minutes, after which the cells were fixed in 100% methanol. To visualize intact and lysed vacuoles the cultures were stained using Diff-Quik (Dade-Behring) according to the manufacturer's instructions. Percent egress was determined by dividing the number of lysed vacuoles by the total number of vacuoles for a sample. For purfalcamine experiments intracellular parasites were treated with purfalcamine (at 0.25, 0.5, 2.5, 25, 50 µM) in HBSS for 15 minutes at 37°C before adding an equal volume of HBSS with purfalcamine and 2 µM A23187 (for a final concentration of 1 µM) for 2, 3, 5 or 10 minutes. Cultures were fixed, stained and analyzed as described above.

### Quantification of ionophore-induced death

Freshly lysed parasites were incubated in serum free DMEM containing 1 µM A23187 or an equivalent amount of DMSO as a solvent control for 45 minutes at a concentration of 1×10^5^ parasites/ml. Following the 45 minute incubation, 2,000 of the treated parasites for each of the time points were then added directly to triplicate wells of a 24-well tissue culture plate containing confluent HFFs in normal culture medium. Plates were incubated for 5 days at 37°C, after which the cells were fixed with 100% methanol and stained with crystal violet to visualize plaques. The number of plaques per well was counted and the percentage survival was determined by dividing the number of plaques formed at each time point in the presence of ionophore by the number of plaques formed in the solvent control well for the equivalent time point.

### Complementation with TgCDPK3 cDNA

The TgCDPK3 coding sequence was amplified from RH parasites cDNA with the sense primer 5′-gcggggccatggggtgcgtccaaga-3′ and the antisense primer 5′-ttaattaatcacgcgtagtccgggacgtcgtacgggtagtgcttcactttgacgtcgca-3′. This primer set introduces an *Nco*I site at the 5′end (single line) and an HA epitope (double line) and a *Pac*I (thick line) at the 3′end. This fragment was digested with *Nco*I and *Pac*I and cloned into the equivalent restriction sites of plasmid pHEX2 [55] to generate pSagCDPK3::HA. This results in the TgCDPK3 coding sequences being between the *T. gondii* Sag1 promoter and 5′UTR and the tubulin 3′UTR.

To generate the different amino acid changes in the TgCDPK3 cDNA we used the QuikChange site-directed mutagenesis kit (Stratagene) to introduce the wanted mutations in pSagCDPK3::HA. The primers used were 5′-gaaggagcgccttggcaTagcctactacattgc-3′ and 5′-gcaatgtagtaggctAtgccaaggcgctccttc-3′ for the TgCDPK3 T239I mutant, and 5′-gagtatgcatgccatggCgGCcgtccactccaagaatc-3′ and 5′-gattcttggagtggacgGCcGccatggcatgcatactc-3′ for the TgCDPK3 G2A,C3A mutant. The bases in upper case are those that are mutated in relation to the wild-type sequence.

### Western blot analysis

Extracellular parasites were resuspended in equal volumes of cytoskeletal protein extract buffer and 2× SDS-PAGE sample buffer (250 mM Tris, pH 6.8, 2% SDS, 20% glycerol, 0.05% bromophenol blue, 600 mM 2-mercaptoethanol) and boiled for 5 minutes. Proteins from approximately 1 million parasite equivalents were separated on a 4–20% SDS page gel and were transferred to a nitrocellulose membrane using standard methods. The membrane was probed with a mouse anti-HA tag monoclonal antibody (Cell Signaling Technology) followed by a peroxidase-conjugated goat anti-mouse antibody (Sigma). The membrane was incubated with SuperSignal West Femto Chemiluminescent Substrate (Pierce) and was used to expose X-Omat Blue film (Kodak).

### Immunofluorescence assays

Immunofluorescence assays (IFA) were performed as described previously [19] using rabbit, anti-HA monoclonal antibodies (Rockland Immunochemical Cell Signaling Technologies) in combination with Alexa fluor-594 or Alexa fluor-488 goat anti-rabbit secondary antibodies (Molecular Probes). Slides were viewed and images were captured with a 100× objective lens on a Nikon Eclipse E100080i microscope, and images. Images were captured using a Hamamatsu C4742-95 camera. The NIS Elements deconvolution software was used to generate images.

### Tagging of endogenous TgCDPK3

The endogenous *CDPK3* locus was hemagglutinin (HA)-tagged using the method described by Huynh and Carruthers [26]. Briefly, a 1.4 kb genomic DNA fragment, which includes the region of *CDPK3* immediately preceding the stop codon, was amplified by PCR using the primers, 5′- TACTTCCAATCCAATTTAgcgtcgggatcaagacacttcc-3′ and 5′- TCCTCCACTTCCAATTTTAGCgtgcttcactttgacgtcgc-3′. In addition to containing sequences for amplifying *CDPK3*, these primers also contain 5′ ligation-independent cloning (LIC) sequences (underlined), which allow for the subsequent ligation-independent cloning of the PCR product. The PCR insert was cloned into the p3XHA9-LIC-DHFR vector (a gift from Vern Carruthers) using previously described methods [26]. The resulting plasmid, pCDPK3-3XHA9-LIC-DHFR was linearized with *Msc*I, which cuts within the *CDPK3* genomic fragment, and was transformed into a *T. gondii* strain, RH*Δku80Δhpt*, which has a decreased rate of non-homologous recombination [25], [26]. Transformed parasites were treated with 1 µM pyrimethamine to select for the presence of the construct. Following selection, the parasites were cloned by limiting dilution, and the clones were tested for the presence of the HA-tag by immunofluorescence using a mouse anti-HA tag monoclonal antibody (Cell Signaling Technology) in conjunction with an Alexa Fluor 594-conjugated goat anti-mouse secondary antibody.

### Cloning and expression of recombinant TgCDPK3recombinant TgCDPK3

Recombinant TgCDPK3 was amplified with Phusion polymerase (New England Biolabs) from the previously published codon optimized expression construct [30], [32] and cloned into the pET28a vector (Novagen) using the Cold-Fusion enzyme kit (Biocat) with the primers 5′-agcggcctggtgccgcgcggcagcggctgcgtgcacagcaaaaatccgc-3′ and 5′-tggtggtggtggtgctcgagtcagtgtttgacttttacatcatcgcaaattt -3′ to generate a N-HIS-tagged TgCDPK3.

Point mutations were introduced by site -directed mutagenesis according to the Phusion protocol. The proteins were expressed in BL21-Rosetta (DE3)pLysS cells (EMD Chemicals) at 18°C and purified using Ni-NTA agarose according to the manufacturer's protocol.

### Kinase activity assays

Activity measurements of recombinant kinase were performed using phosphorylation of Syntide-2 and subsequent scintillation counting. 100 nM recombinant CDPK3 were pre-incubated at 37°C in a kinase reaction mix (New England Biolabs) containing 100 µM CaCl_2_ and 100 µM ATP to allow auto-phosphorylation in a final reaction volume of 25 µL at 30 degrees Celsius. After 5 minutes the reaction was supplemented to a final concentration of 1 mM Syntide-2 and 2.5 µCi ^32^[P]ATP and incubated at 30°C for 8 minutes. To test the effect of purfalcamine recombinant kinase was preincubated with CaCl_2_ and ATP in the presence of drug or DMSO for 5 minutes prior to addition of syntide-2 and ^32^[P]ATP. The reactions were stopped by the addition of phosphoric acid to 75 mM and half the reaction spotted onto P81 phosphocellulose squares (Millipore), air dried and subsequently washed 3 times for 2 minutes in 75 mM phosphoric acid. Syntide 2-phosphorylation was measured using a scintillation counter.

### Generation of Tgcdpk3 knockout strain

A knockout construct was generated using the pminiGFP.ht vector [19], which contains the *T. gondii HPT* gene flanked two multiple cloning sites. To design the knockout construct, first a PCR fragment amplified with primers 5′-ggggtaccagccgtatctcgtagcgaataaac-3′ and 5′-ggaagcttcgtgacttctacagatttgcgtct-3′ was digested with *Kpn*I and *Hind*III and cloned into pminiGFP.ht digested with the same restriction enzymes. The resulting plasmid was digested with *Not*I and *Xba*I ligated to a PCR fragment amplified with primers 5′-aagggaaaagcggccgcgcaggttccctctggtggtgtactc-3′ and 5′-gagggtcagttcctccaagcctccc-3′ and also digested with *Not*I and *Xba*I.

The resulting vector, pKOCDPK3, was linearized with *Not*I and 30 µg of DNA were introduced into RHΔ*hpt*Δ*ku80* strain [25], [26] by standard methods [56]. Parasites were then grown in T25 flasks containing HFFs in normal culture medium for 24 hours at which point the media was changed to culture medium containing 50 µg/ml MPA and 50 µg/ml xanthine. After parasites stably expressing HPT were established, the parasites were cloned by limiting dilution. 12 clones were tested by PCR for the desired disruption using genomic DNA isolated with the DNeasy Blood and Tissue Kit (QIAGEN).

### Invasion assay

The efficiency of invasion was determined by allowing 2×10^6^ parasites to invade confluent HFFs grown on coverslips for 15, 30, 45 or 60 minutes. To differentiate those parasites that have entered cells from those that remained outside we utilized an immunofluorescence-based invasion assay. In summary, following fixation with 4% formaldehyde, external parasites were labeled with an antibody against the parasite surface generated in rabbits (Abcam Labs). Cells were then permeabilized with 0.2% Triton X-100 in PBS, and all parasites were labeled with a second SAG1 antibody that had been generated in mice (a gift from Dr. Peter Bradley). The two primary antibodies were visualized with an anti-rabbit IgG secondary antibody with a red fluorescent tag (Alexa Fluor 594 - Molecular Probes) and an anti-mouse IgG secondary antibody with a green fluorescent tag (Alexa Fluor 488 - Molecular Probes) respectively. Thus external parasites were co-labeled red and green, while intracellular parasites were labeled green only. In this manner we determined the number of intracellular parasites in 20 randomly chosen fields of view for each coverslip using a Zeiss Axiovert 40 CFL microscope (400× magnification). The data was expressed as the percentage of parasites detected in 20 randomly selected fields of view that are inside cells.

### Motility assay

Cover slips were coated with 0.1% (w/v) poly-l-lysine in water (Sigma-Aldrich) for 10 minutes and washed with PBS, then dried out at 37°C for 1 hour. A total of 1×10^5^ freshly lysed parasites were allowed to settle down on the poly L-lysine coated glass for 20 minutes in intracellular (IC) buffer (5.8 mM NaCl, 141.8 mM KCl, 1 mM CaCl_2_, 1 mM MgCl_2_, 5.6 mM glucose and 25 mM Hepes, pH 7.2) [57]. Media was then changed to extracellular (EC) buffer (141.8 mM NaCl, 5.8 mM KCl, 1 mM CaCl_2_, 1 mM MgCl_2_, 5.6 mM glucose, 25 mM *N*-2-hydroxyethylpiperazine-*N′*-2-ethanesulfonic acid (Hepes)-NAOH, pH 7.2 [43]) and parasites were incubated at 37°C for 15 minutes. After fixation with 3% formaldehyde, samples were stained with antibodies against SAG1 using standard IFA protocols [19]. The level of motility in each treatment was determined through fluorescence microscopy by counting the number of parasites on each cover slip that were associated with a Sag1 trail in 10 randomly selected fields.

To supplement EC buffer we added KCl (37 mM final concentration), MgCl_2_ (8 mM final concentration), MnCl_2_ (10 mM final concentration), CaCl_2_ (10 mM final concentration) or CsCl (30 mM final concentration).

### Quantitation of division rate

To compare rate of division of the two strains, 1×10^5^ parasites were allowed to invade confluent HFFs for 2 hours in a 24 well plate. Wells were then washed 2 times in PBS, and re-filled with normal culture medium. 24 hours after parasite invasion, the cells were fixed in 4% formaldehyde, and parasites stained using an antibody directed against SAG1 as previously described [58]. The number of parasites per vacuole for a minimum of 100 randomly chosen vacuoles was then counted for each strain using a Nikon Eclipse 2000-5 microscope at 1000× magnification.

## Supporting Information

Figure S1 Position of mutated amino acids in TgCDPK3. A. Protein sequence of TgCDPK3. The glycine mutated in 52F11 (G88) is highlighted in black. The leucine mutated in MBD1.1(L184) is highlighted in dark gray. The asparagine mutated in 91E4 is boxed. The threonine mutated in MBE1.1 (T239) is highlighted in light gray. The HRD domain is double underlined. The region of the protein equivalent to subdomain VIII found in members of the protein kinase superfamily is underlined. B. Alignment of the T loop of the activation domain from all the CDPKs found in both Toxoplasma gondii and Plasmodium falciparum. The arrow points at the Thr mutated in MBE1.1. Alignment was performed by Clustal W using the whole protein sequences of all listed proteins. Nomenclature is based on Billker O, Lourido S, Sibley LD (2009), Cell Host Microbe 5: 612–622.
(DOC)

Figure S2 Effect of TgCDPK3 mutation on splicing and transcript level. A. The distribution of exons (white boxes) and introns (dashed lines) along the TgCDPK3 genomic locus are shown in the diagram. Numbers indicate amount of bases in genomic DNA with 1 representing the putative transcription start for TgCDPK3. Black arrowhead indicates translation start site, the white arrowhead points at the relative position of the stop codon and the arrow points at the relative position of the mutation in MBE1.1. The sequence around the junction of exon 3 (capital letters) and intron 3 (lower cap letters) is shown for both the parental and mutant strains with the SNP in gray box. B. Sequence chromatogram of fragments of TgCDPK3 obtained by PCR from cDNA or through 3′RACE. Vertical line indicates the junction of exons 3 and 4 of TgCDPK3.
(TIF)

Figure S3 Real-time quantitative PCR analysis of CDPK3 transcript abundance in MBE1.1. RNA was extracted from intracellular MBE1.1 and parental strain parasites, and the relative abundance of the CDPK3 transcript was measured using the comparative Ct real-time PCR method. Each bar represents the average of three independent experiments. The error bars represent one standard deviation.
(TIF)

Figure S4 Phenotypic complementation of MBE1.1. A. The diagram shows the arrangement of the transgenic copy of wild-type TgCDPK3 used in the complementation experiments. The coding sequence (box) contains the TgCDPK3 ORF and an HA tag (in gray) and is driven by the 4.1 kb genomic region upstream of the TgCDPK3 start codon (CDPK3ups). The 3′UTR is form the T. gondii Tubulin (tub) transcript. B. Intracellular parasites of the parental (RHΔhpt), mutant (MBE1.1) and complemented (MBE1.1+CDPK3HA) strains where exposed to 1 µM A23187 for the time points indicated in the graph. Percentage egress represents the number of lysed vacuoles divided by the total number of vacuoles (lysed+intact). Each data point represents the average of our independent experiments and the error bars represent the standard deviation. At least 100 vacuoles were counted per data point. C. Protein levels of exogenous TgCDPK3 and TgCDPK3(G2A,C3A) expressed in the MBE1.1 mutant strain either by the Sag promoter or the CDPK3 promoter. Equal amount of protein was loaded for all samples and Sag1 antibody was used to confirm equal loading. Exogenous TgCDPK3 was detected with antibodies against the HA tag.
(TIF)

Figure S5 Mutations in the TgCDPK3 locus in strains MBD1.1, 52F11 and 91E4. DNA sequence chromatograms of bases 4,684,043-52 of chromosome IX in mutant MBD1.1 (A), bases 4,683,200-7 in mutant 52F11 (B) and bases 4,684,596 to 4,684,600 in 91E4 (C) in comparison with the same regions of chromosome IX in the parental strains RHΔhpt (MBD1.1) or PruΔhpt (52F11 and 91E4). Gray box highlights the SNP found in the mutant strain (A, B and C).
(TIF)

Figure S6 Sequences and relative position of primers used to generate fragments of TgCDPK3 for sequencing of genomic locus.
(TIF)
