# Supplementary material for: A Forward Genetic Screen Reveals that Calcium-dependent Protein Kinase 3 Regulates Egress in Toxoplasma
Source: PLoS Pathog. 2012 Nov 29;8(11):e1003049. doi: 10.1371/journal.ppat.1003049 (PMC3510250; doi:10.1371/journal.ppat.1003049)
Supplement: Figure S1 — Position of mutated amino acids in TgCDPK3. A. Protein sequence of TgCDPK3. The glycine mutated in 52F11 (G88) is highlighted in black. The leucine mutated in MBD1.1(L184) is highlighted in dark gray. The asparagine mutated in 91E4 is boxed. The threonine mutated in MBE1.1 (T239) is highlighted in light gray. The HRD domain is double underlined. The region of the protein equivalent to subdomain VIII found in members of the protein kinase superfamily is underlined. B. Alignment of the T loop of the activation domain from all the CDPKs found in both Toxoplasma gondii and Plasmodium falciparum. The arrow points at the Thr mutated in MBE1.1. Alignment was performed by Clustal W using the whole protein sequences of all listed proteins. Nomenclature is based on Billker O, Lourido S, Sibley LD (2009), Cell Host Microbe 5: 612–622. (DOC) [file ppat.1003049.s001.doc]

MGCVHSKNPH SKHAGAAGEK PDASLEKGGQ SKGSAPSSGT GDSGKGTGSP 50 DTKRDSMPMT PGMYITQQKA HLSDRYQRVK KLGSGAYGEV LLCKDKLTGA 100 ERAIKIIKKS SVTTTSNSGA LLDEVAVLKQ LDHPNIMKLY EFFEDKRNYY 150 LVMEVYRGGE LFDEIILRQK FSEVDAAVIM KQVLSGTTYL HKHNIVHRDL 200 KPENLLLESK SRDALIKIVD FGLSAHFEVG GKMKERLGTA YYIAPEVLRK 250 KYDEKCDVWS CGVILYILLC GYPPFGGQTD QEILKRVEKG KFSFDPPDWT 300 QVSDEAKQLV KLMLTYEPSK RISAEEALNH PWIVKFCSQK HTDVGKHALT 350 GALGNMKKFQ SSQKLAQAAM LFMGSKLTTL EETKELTQIF RQLDNNGDGQ 400 LDRKELIEGY RKLMQWKGDT VSDLDSSQIE AEVDHILQSV DFDRNGYIEY 450 SEFVTVCMDK QLLLSRERLL AAFQQFDSDG SGKITNEELG RLFGVTEVDD 500 ETWHQVLQEC DKNNDGEVDF EEFVEMMQKI CDVKVKH 537

**A.**

**B.**
