# Supplementary figures and images for: A Forward Genetic Screen Reveals that Calcium-dependent Protein Kinase 3 Regulates Egress in Toxoplasma
Source: PLoS Pathog. 2012 Nov 29;8(11):e1003049. doi: 10.1371/journal.ppat.1003049 (PMC3510250; doi:10.1371/journal.ppat.1003049)

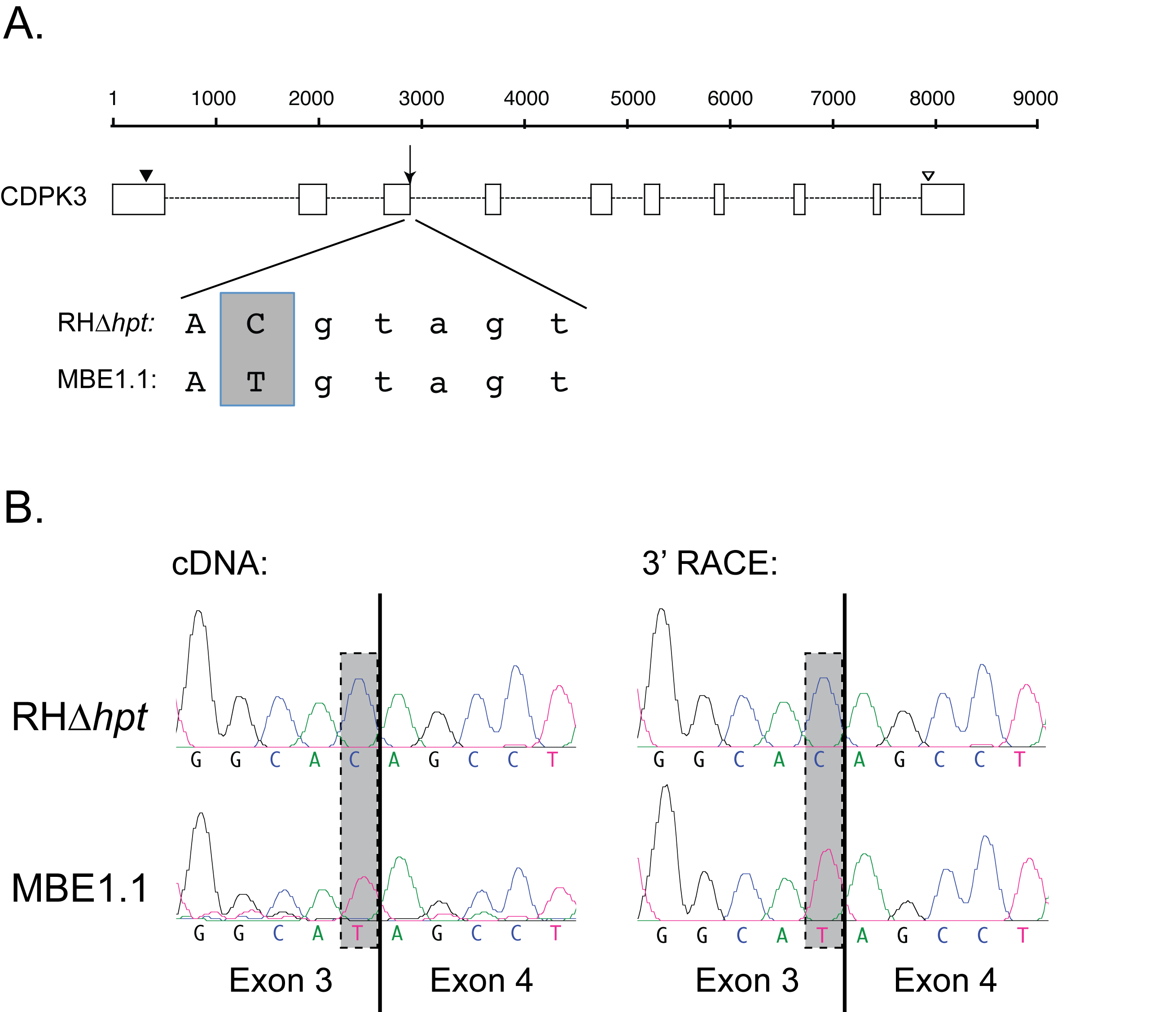

Supplement: Figure S2 — Effect of TgCDPK3 mutation on splicing and transcript level. A. The distribution of exons (white boxes) and introns (dashed lines) along the TgCDPK3 genomic locus are shown in the diagram. Numbers indicate amount of bases in genomic DNA with 1 representing the putative transcription start for TgCDPK3. Black arrowhead indicates translation start site, the white arrowhead points at the relative position of the stop codon and the arrow points at the relative position of the mutation in MBE1.1. The sequence around the junction of exon 3 (capital letters) and intron 3 (lower cap letters) is shown for both the parental and mutant strains with the SNP in gray box. B. Sequence chromatogram of fragments of TgCDPK3 obtained by PCR from cDNA or through 3′RACE. Vertical line indicates the junction of exons 3 and 4 of TgCDPK3. (TIF) [file ppat.1003049.s002.tif]

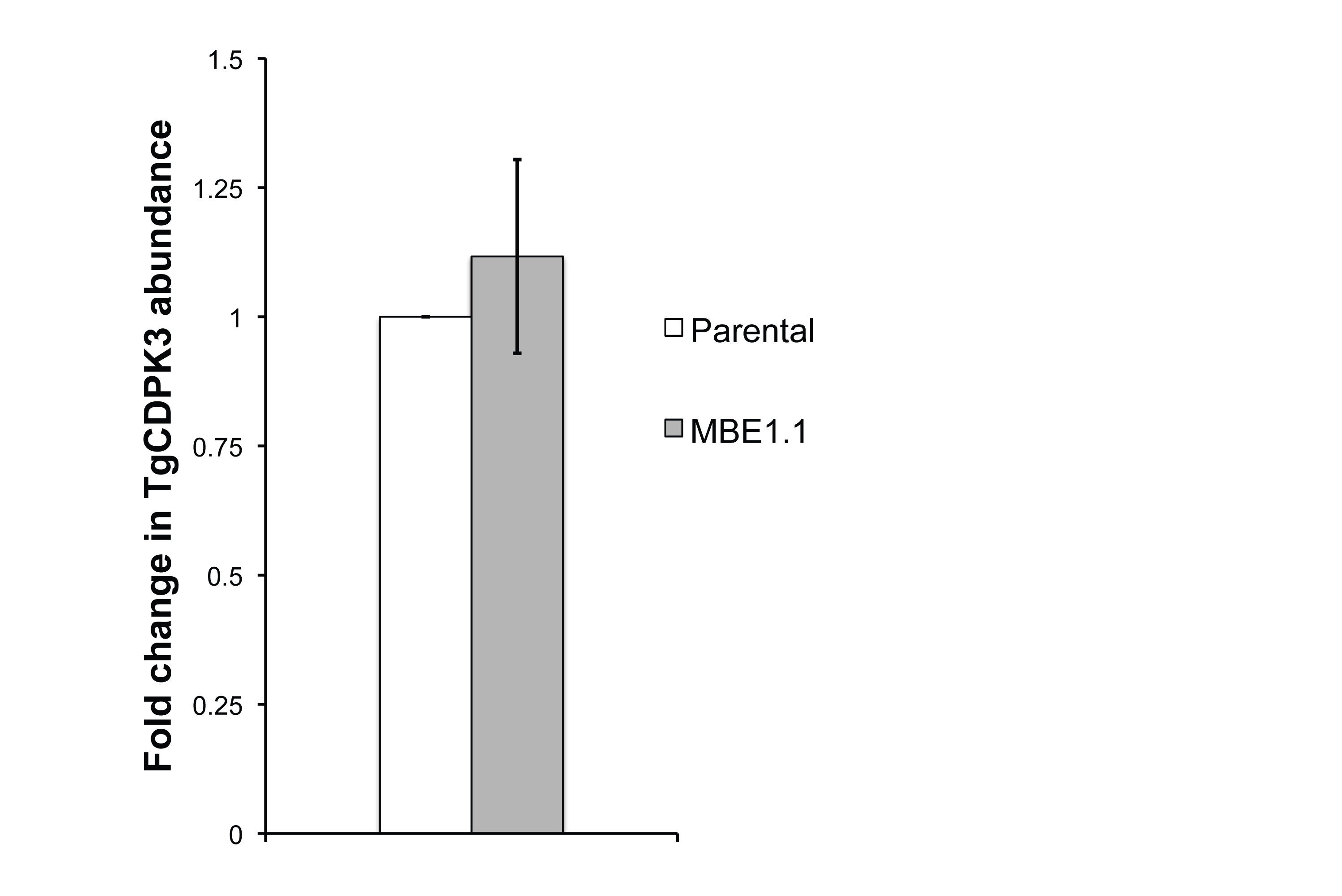

Supplement: Figure S3 — Real-time quantitative PCR analysis of CDPK3 transcript abundance in MBE1.1. RNA was extracted from intracellular MBE1.1 and parental strain parasites, and the relative abundance of the CDPK3 transcript was measured using the comparative Ct real-time PCR method. Each bar represents the average of three independent experiments. The error bars represent one standard deviation. (TIF) [file ppat.1003049.s003.tif]

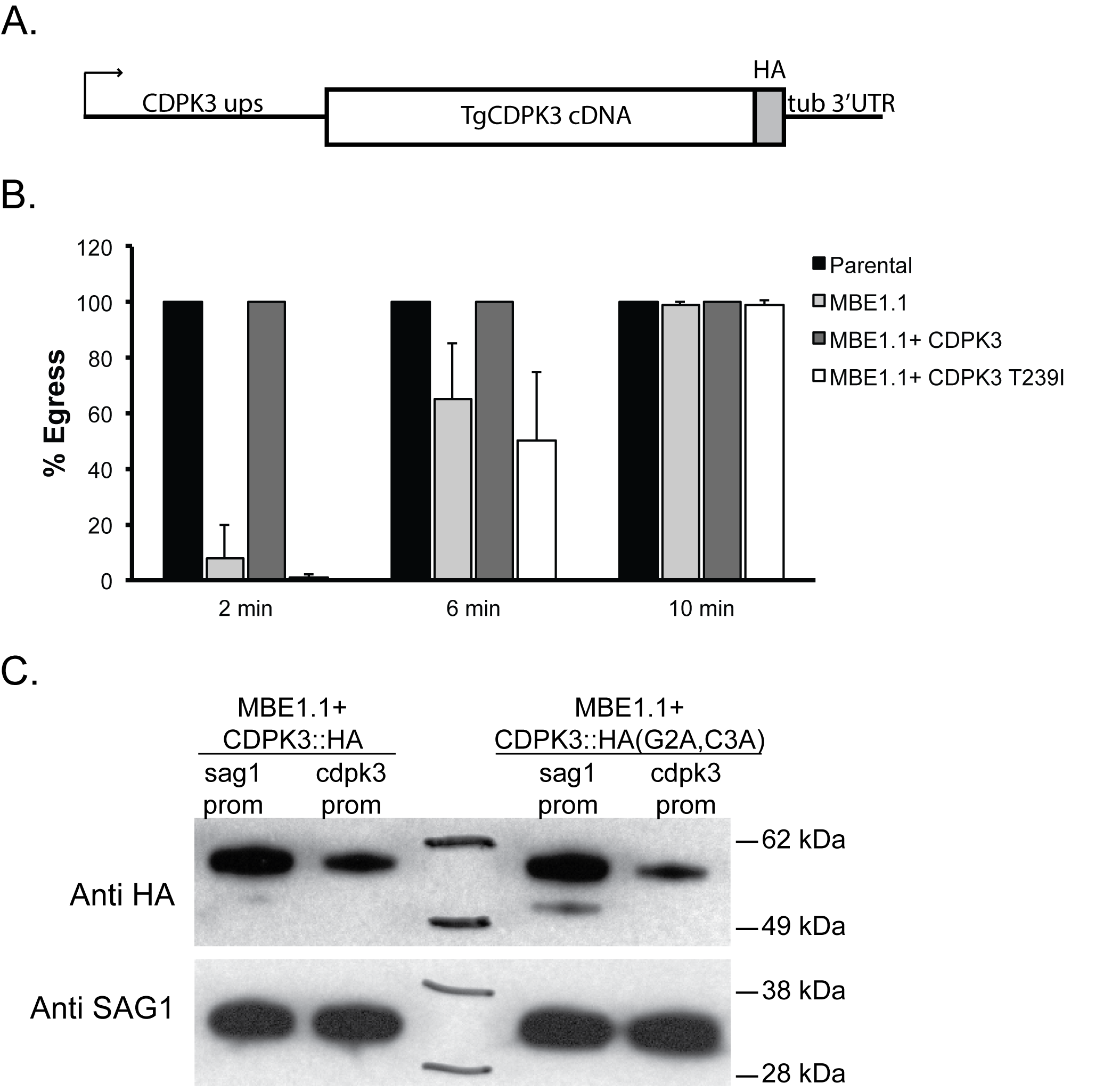

Supplement: Figure S4 — Phenotypic complementation of MBE1.1. A. The diagram shows the arrangement of the transgenic copy of wild-type TgCDPK3 used in the complementation experiments. The coding sequence (box) contains the TgCDPK3 ORF and an HA tag (in gray) and is driven by the 4.1 kb genomic region upstream of the TgCDPK3 start codon (CDPK3ups). The 3′UTR is form the T. gondii Tubulin (tub) transcript. B. Intracellular parasites of the parental (RHΔhpt), mutant (MBE1.1) and complemented (MBE1.1+CDPK3HA) strains where exposed to 1 µM A23187 for the time points indicated in the graph. Percentage egress represents the number of lysed vacuoles divided by the total number of vacuoles (lysed+intact). Each data point represents the average of our independent experiments and the error bars represent the standard deviation. At least 100 vacuoles were counted per data point. C. Protein levels of exogenous TgCDPK3 and TgCDPK3(G2A,C3A) expressed in the MBE1.1 mutant strain either by the Sag promoter or the CDPK3 promoter. Equal amount of protein was loaded for all samples and Sag1 antibody was used to confirm equal loading. Exogenous TgCDPK3 was detected with antibodies against the HA tag. (TIF) [file ppat.1003049.s004.tif]

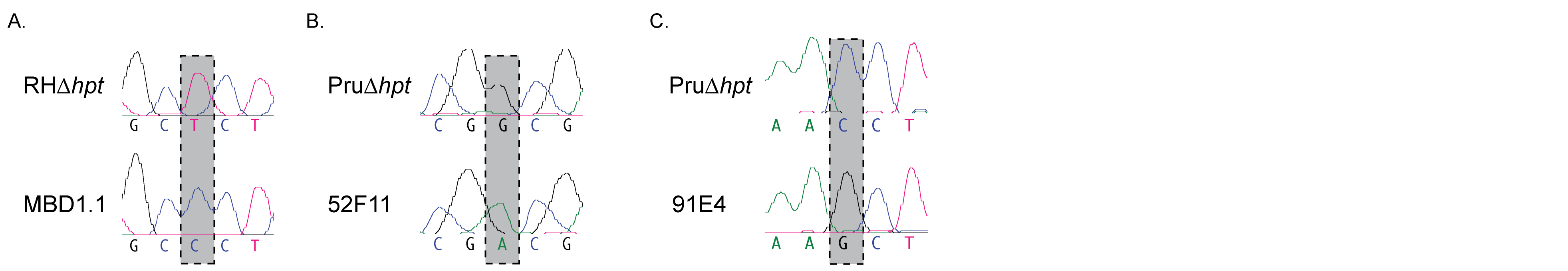

Supplement: Figure S5 — Mutations in the TgCDPK3 locus in strains MBD1.1, 52F11 and 91E4. DNA sequence chromatograms of bases 4,684,043-52 of chromosome IX in mutant MBD1.1 (A), bases 4,683,200-7 in mutant 52F11 (B) and bases 4,684,596 to 4,684,600 in 91E4 (C) in comparison with the same regions of chromosome IX in the parental strains RHΔhpt (MBD1.1) or PruΔhpt (52F11 and 91E4). Gray box highlights the SNP found in the mutant strain (A, B and C). (TIF) [file ppat.1003049.s005.tif]
